# Neurovascular Coupling in Development and Disease: Focus on Astrocytes

**DOI:** 10.3389/fcell.2021.702832

**Published:** 2021-07-12

**Authors:** Teresa L. Stackhouse, Anusha Mishra

**Affiliations:** ^1^Department of Neurology, Jungers Center for Neurosciences Research, Oregon Health & Science University, Portland, OR, United States; ^2^Knight Cardiovascular Institute, Oregon Health & Sciences University, Portland, OR, United States

**Keywords:** neurovascular unit, neurovascular coupling, astrocytes, cerebrovascular development, cerebrovascular dysfunction, stroke, Alzheimer’s disease

## Abstract

Neurovascular coupling is a crucial mechanism that matches the high energy demand of the brain with a supply of energy substrates from the blood. Signaling within the neurovascular unit is responsible for activity-dependent changes in cerebral blood flow. The strength and reliability of neurovascular coupling form the basis of non-invasive human neuroimaging techniques, including blood oxygen level dependent (BOLD) functional magnetic resonance imaging. Interestingly, BOLD signals are negative in infants, indicating a mismatch between metabolism and blood flow upon neural activation; this response is the opposite of that observed in healthy adults where activity evokes a large oversupply of blood flow. Negative neurovascular coupling has also been observed in rodents at early postnatal stages, further implying that this is a process that matures during development. This rationale is consistent with the morphological maturation of the neurovascular unit, which occurs over a similar time frame. While neurons differentiate before birth, astrocytes differentiate postnatally in rodents and the maturation of their complex morphology during the first few weeks of life links them with synapses and the vasculature. The vascular network is also incomplete in neonates and matures in parallel with astrocytes. Here, we review the timeline of the structural maturation of the neurovascular unit with special emphasis on astrocytes and the vascular tree and what it implies for functional maturation of neurovascular coupling. We also discuss similarities between immature astrocytes during development and reactive astrocytes in disease, which are relevant to neurovascular coupling. Finally, we close by pointing out current gaps in knowledge that must be addressed to fully elucidate the mechanisms underlying neurovascular coupling maturation, with the expectation that this may also clarify astrocyte-dependent mechanisms of cerebrovascular impairment in neurodegenerative conditions in which reduced or negative neurovascular coupling is noted, such as stroke and Alzheimer’s disease.

## Neurovascular Coupling

The brain is an energy-hungry organ; it comprises only 2% of body weight but uses about 20% of the body’s resting energy (Attwell and Laughlin, 2001; Howarth et al., 2012). This high energy demand is met by a constant supply of energy substrates delivered via extensive cerebral vascular networks (Blinder et al., 2013). An increase in the metabolic demand of neurons during activity induces a further increase in cerebral blood flow (CBF): this coupling between neuronal activity and CBF is termed neurovascular coupling (NVC). NVC is a fundamental aspect of healthy brain function and underlies the basis for non-invasive brain imaging techniques used in clinical and research settings. Such techniques, including blood oxygen level dependent (BOLD) functional magnetic resonance imaging (fMRI) (Howarth et al., 2021), positron emission tomography (Cecchin et al., 2021), and the more recent functional ultrasound (Mace et al., 2011), use changes in CBF or the subsequent changes in energy substrates as a proxy for neuronal activity. Research in the last two decades has taught us much about the cellular mechanisms underlying NVC. However, these mechanisms are not yet fully characterized. Notably, neural activity does not reliably correlate with increases in CBF in the developing brain in rodent models or humans, nor in the context of neurodegenerative diseases. Despite their importance, the mechanisms underlying reduced NVC in development and disease are understudied. In this review, we introduce the components of the neurovascular unit (NVU), briefly summarize known signaling mechanisms that mediate NVC, describe the changes that occur in NVC and NVU components during development and during neurological disorders, and end by suggesting future research directions to elucidate the mechanisms that underpin these NVC changes. We pay particular attention to astrocytes, the major glial component of the NVU that contributes to NVC. Similarities between immature astrocytes in the developing brain and reactive astrocytes in disease contexts, including in ischemic stroke and Alzheimer’s disease (AD), suggest parallel astrocyte-dependent pathways may underlie reduced NVC in both development and disease. Thus, a comprehensive understanding of how NVC is regulated during development and neurological disorders will not only improve our understanding of these biologically important processes, but potentially also guide new therapeutic interventions in disease conditions characterized by cerebrovascular dysfunction.

## Neurovascular Unit: Components and Development

The NVU is an integrated and interactive entity comprised of neurons, glia (astrocytes and microglia), and cerebral vascular cells (vascular smooth muscle cells (VSMCs), pericytes, and endothelial cells). The NVU is important for maintaining the blood-brain barrier (BBB), a structure crucial for maintaining brain homeostasis that tightly controls the transport of molecules and immune cells into and out of the central nervous system (CNS) (Abbott et al., 2010). Breakdown of the BBB is evident in disease and results in exposure of brain tissue to harmful blood components such as fibrinogen and albumin (Schachtrup et al., 2010; Weissberg et al., 2015), which can exacerbate or cause neurological dysfunction. NVU components are important for BBB establishment and maintenance. This is extensively reviewed elsewhere (Sweeney et al., 2019). In this review, we focus on the role of the NVU components in activity-dependent changes in CBF, that is, NVC.

Changes in NVC manifested during development and NVU maturation may explain conflicting findings reported by numerous groups. During development, the vasculature, neuronal circuits, and astrocyte networks must integrate to form the NVU, maintain a healthy BBB, and engage NVC mechanisms. The complete NVU is closely linked in activity beginning in the early stages of development and into adulthood.

Though neural cells and the vasculature have distinct embryonic origins, they have strikingly intertwined time courses of proliferation, migration, and terminal differentiation, and a common array of signaling molecules that regulate both brain and vascular development (Raab and Plate, 2007; Walchli et al., 2015b). Even in the adult brain, neurogenesis occurs in regions of angiogenesis, suggesting a close relationship between the two (Palmer et al., 2000). Indeed, vascular endothelial cells synthesize brain-derived neuronal growth factor (BDNF) to precisely guide the migration of adult neuronal progenitors from the subventricular zone to the olfactory bulb (Snapyan et al., 2009). The relationship between neural and vascular development is not just limited to proliferation cues. Early (P0-P5) in mouse barrel cortex development, neural activity drives vascular formation and patterning (Lacoste et al., 2014). Thus, vasculature-derived signals can drive proper neuronal migration and positioning, while neural activity can, conversely, drive proper branching and vascular patterning.

NVU development further depends on reciprocal feedback signaling between blood vessels and astrocytes. Astrocytes are required for proper blood vessel density and branching throughout development, and inhibition of astrogliogenesis leads to a significant decrease in vessel density and branching in both the cortex (Ma et al., 2012) and the retina (O’Sullivan et al., 2017). Thus, astrocyte and cortical vessel development coincide and are interdependent, and the mechanisms driving NVC could also vary as the system matures. Microglia also play a role in vasculogenesis (Arnold and Betsholtz, 2013; Reemst et al., 2016) and use blood vessels as migration highways in the developing brain (Mondo et al., 2020). Although the complete NVU is not fully established for the first few weeks after birth in rodents, neuronal activity can regulate cerebral vessels via astrocytes as early as P9 (Zonta et al., 2003). Apparent morphological maturation of the NVU occurs by the third week of life (Coelho-Santos and Shih, 2020); however, the timeline over which functional NVC mechanisms mature is not fully elucidated.

### Neurons

The stages of brain development on the level of neurons have been very well characterized in humans and rodents. The major timeframe within which neurogenesis occurs in rodents is embryonic day 9.5 (E9.5) to postnatal day 15 (P15) (Altman and Bayer, 1990a,b; Letinic et al., 2002; Sillitoe and Joyner, 2007; Sudarov and Joyner, 2007), while the comparable period in humans is ∼10–28 gestational weeks (Letinic et al., 2002). Synaptogenesis and myelination occur after birth and continue throughout adolescence, coinciding with the generation and growth of astrocytes and oligodendrocytes, respectively. In the case of myelin, this process continues into adulthood (Andersen, 2003; Watson et al., 2006; Semple et al., 2013).

Neurons are integral within the NVU as the driving agents initiating increases in CBF during functional hyperemia, and as the cells that consume most of the energy used by the brain (Howarth et al., 2021). Neurons interact with astrocytes, microglia, and blood vessels, and their communication at each of these interfaces influences how the NVU responds to neural activity to induce NVC and mediate changes in CBF (Attwell et al., 2010). When research into NVC first began, it was believed to be a process mediated by feedback regulation, such that a decrease in energy substrates signaled an increase in blood flow for energy production (Attwell et al., 2010). However, a lack of oxygen and glucose, the primary substrates necessary for adenosine triphosphate (ATP) production via glycolysis and oxidative phosphorylation, does not fully explain the hemodynamic response, as the response persists even under conditions wherein both substrates are present at high concentrations (Powers et al., 1996; Wolf et al., 1997; Lindauer et al., 2010). Instead, these data support the notion that NVC is engaged in a feed-forward manner wherein, regardless of the available energy substrates, CBF is increased (at least in adult animals) following synaptic activity via intercellular signaling between NVU components (reviewed in Arthurs et al., 2000; Attwell et al., 2010; Mishra, 2017). This feed-forward process likely ensures an oversupply of substrates to preemptively prohibit a situation where neural tissue might receive an insufficient energy supply (Leithner et al., 2010; Buxton, 2021). Indeed, even when several identified NVC signaling pathways are inhibited in combination, this response cannot be entirely blocked (Liu et al., 2012; Hosford and Gourine, 2019). It is also possible that the large increase in blood flow relative to the metabolic needs of the tissue exists to wash away metabolic by-products such as carbon dioxide and lactate or is a result of the consequent acidosis. However, several papers suggest that pH in the extracellular space remains constant despite increases in lactate (Ueki et al., 1988) or even rises due to the activity of plasma membrane Ca^2+^-ATPases (Makani and Chesler, 2010) and extracellular carbonic anhydrase (Chen and Chesler, 1992). Furthermore, preventing acidosis did not reduce neurovascular coupling despite reducing vascular reactivity to hypercapnia (Liu et al., 2012). Another potential but under-examined reason for NVC may be that the increased blood flow serves as a sink for the heat generated during intense brain activity. In our view, the persistence of NVC stresses its importance for healthy brain function and underscores the myriad parallel and compensatory mechanisms in place to maintain it. It also strongly indicates that the pathways underlying NVC have not been comprehensively elucidated.

Neuronal activity can trigger local changes in CBF directly by releasing vasoactive molecules onto arterioles to engage VSMCs or indirectly via signaling to astrocytes, which then release vasoactive molecules onto capillary pericytes (details to be discussed below; Biesecker et al., 2016; Mishra et al., 2016; Mishra, 2017). Neuron-derived vasoactive molecules include cyclooxygenase-2-derived prostanoids (Niwa et al., 2000a) and nitric oxide (NO) (Dirnagl et al., 1993; Akgören et al., 1994, 1996; Gotoh et al., 2001; Lourenço et al., 2014; Mapelli et al., 2017; Echagarruga et al., 2020). The relative contributions of excitatory and inhibitory neurons to NVC is still a field of intense research (reviewed by Howarth et al., 2021), with current evidence supporting a potentially more important and predominant role for inhibitory neurons, particularly NO synthase-positive interneurons (Echagarruga et al., 2020; Howarth et al., 2021). Projection neurons from subcortical regions such as the basal ganglia (e.g., cholinergic neurons) and brainstem nuclei (e.g., noradrenergic neurons in the locus coeruleus) can also regulate cortical blood flow by mediating widespread change in vascular tone (Hamel, 2006) and modulating local NVC response via their actions on cortical neurons, astrocytes, and the vasculature (Scremin et al., 1973; Cauli and Hamel, 2010; Lecrux et al., 2017; see Lecrux et al., 2019; Howarth et al., 2021 for comprehensive reviews).

### Astrocytes

During development, astrocytes are derived from radial glia and generated after neurons. Astrocyte proliferation rates differ throughout life and in response to injury (Bardehle et al., 2013). During human fetal development, astrocytes are one of the last cells to be generated, and white matter fibrous astrocytes are generated earlier than gray matter protoplasmic astrocytes (Marin-Padilla, 1995). Interestingly, both white and gray matter astrocytes form in parallel with vasculature ingrowth in those regions; these growth patterns are so interconnected they have been described as “inseparable” from each other (Marin-Padilla, 1995). Less is known about astrocyte postnatal development in humans (de Majo et al., 2020), but it is speculated that astrocytes continue to proliferate and cover the vasculature with their endfeet in step with the expanding brain.

**FIGURE 1:**
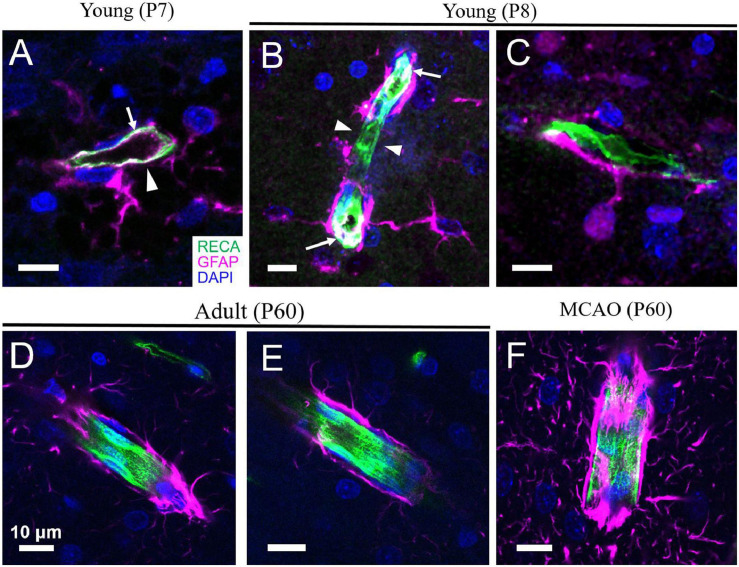
Astrocyte coverage of arterioles during development, adulthood and after stroke. **(A,B)** In the cortex of developing rats, angiogenesis and astrogliogenesis is occurring concurrently. Although astrocytes begin enwrapping the vasculature immediately, this coverage is incomplete (arrowheads). GFAP expression can sometimes also be detected in the endothelium of very young rats (arrows). **(C)** An astrocyte with immature morphology lacking many processes is shown with a primary process extending to form an endfoot on a nearby capillary. **(D,E)** By mature adulthood (P60), astrocyte endfeet coverage of the blood vessels is complete. **(F)** Following middle cerebral artery occlusion (MCAO, a model of ischemic stroke), increased expression of GFAP and thickening of astrocyte endfeet on vessels is evident. Green = rat endothelial cell antigen-1 (RECA-1), magenta = glial fibrillary acidic protein (GFAP), blue = 4’,6-diamidino-2-phenylindole (DAPI). Scale bars = 10 μm. [Panels **D,F** are reproduced, with permission, from McConnell et al. (2019)].

**FIGURE 2:**
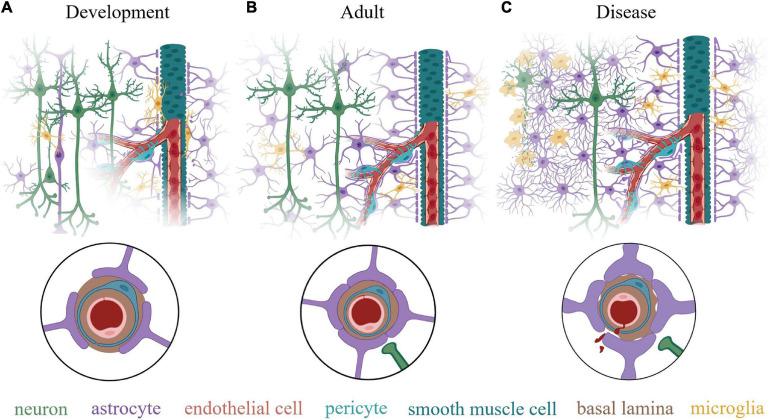
Schematic illustration of the neurovascular unit (NVU) at various life stages. **(A)** During early post-natal development, all the component cells of the NVU are present but the NVU is not yet mature. This stage is characterized by continuing vasculogenesis and astrogliogenesis and an incomplete coverage of the vasculature by astrocyte endfeet. Neuron density and synapse number is high, while synaptic pruning by microglia to tune the neuronal circuits is continuing. Many microglia are present directly on the vessels, which they use as as migration routes while also guiding angiogenesis. Although cerebral vessels grow and develop with an intact barrier function, it is incomplete without full coverage by astrocyte endfeet, which are necessary for blood-brain barrier maintenance. **(B)** In the adult (> at least P21), this system has matured with distinct tiling of astrocytes within the neural tissue and complete endfeet coverage of the cerebral vasculature. An estimated 99.7% of vasculature is covered by astrocyte endfeet in adults, with some spots in contact with microglia. Neuronal density is reduced compared to early development and synaptic pruning is complete. **(C)** In the context of neurological diseases or injuries, degenerating neurons are physically separated from healthy neuronal tissue by an astrocyte glial scar, composed of overlapping processes of reactive astrocytes that proliferate and lose their territorial properties. Microglia are activated, taking on a phagocytic phenotype at the site of tissue damage. Astrocyte endfeet appear thicken due to an increase in GFAP and vimentin expression and may retract from the basal lamina, which diminishes blood-brain barrier integrity and may interfere with neurovascular coupling and paravascular flow. Insets in circles show a cross section of a capillary in each condition. Created with BioRender.com.

In rodents, developmental proliferation of astrocytes occurs mostly within the week prior to and following birth, followed by differentiation and maturation during the first few weeks (Tien et al., 2012). Endfeet formation on the cerebral vasculature begins during the first week after birth alongside astrogliogenesis (Figures 1, 2; Lunde et al., 2015; de Majo et al., 2020). Formation of this gliovascular interface correlates with the expression of many proteins that characterize mature astrocytes and appears to drive their polarization (Ge et al., 2012; Lunde et al., 2015; Farmer et al., 2016; Clavreul et al., 2019). Hallmark features of mature astrocytes, including aquaporin 4 (AQP4), glial fibrillary acidic protein (GFAP), calcium-binding protein S-100β, glutamate-aspartate transporter GLAST, aldehyde dehydrogenase 1A1 (ALDH1A1), and the inward-rectifying K^+^ channel Kir4.1 increase progressively during the first few weeks of life (Shibata et al., 1997; Araque and Navarrete, 2010; Clarke et al., 2018). Expression of Kir4.1 in astrocytes is associated with a shift in their resting membrane potential from −50 to −80 mV, which reflects the reversal potential of K^+^ and is a physiological characteristic of mature astrocytes. Kir4.1 expression is also necessary for loss of proliferative properties and enhanced differentiation of astrocytes (Bordey et al., 2001; Higashimori and Sontheimer, 2007).

Astrocytes are a crucial component of the NVU (McConnell et al., 2019) but, besides this, they also play key roles in synapse formation and function (Christopherson et al., 2005; Allen and Eroglu, 2017). Within the neuropil, astrocytes evenly tile the parenchyma with few overlapping processes but are connected to neighboring astrocytes via gap junctions to form a syncytium (Bushong et al., 2002; Kofuji and Newman, 2004; Tsai et al., 2012). Peri-synaptic processes of astrocytes closely interdigitate and communicate with neurons (Spacek, 1985; Ventura and Harris, 1999; Witcher et al., 2007, 2010). These peri-synaptic astrocyte processes mature alongside the pre- and post-synaptic structures of neurons during early developmental periods and contribute to synaptic plasticity in adulthood (Trotter et al., 2020, preprint). Astrocytes perform multiple specialized housekeeping tasks to maintain healthy brain function, such as K^+^ uptake (Kuffler et al., 1966; Olsen and Sontheimer, 2008), neurotransmitter clearance (Hertz, 1979; Brew and Attwell, 1987) and recycling (Norenberg and Martinez-Hernandez, 1979; Sonnewald et al., 1993; Bak et al., 2006), as well as bicarbonate transport to buffer extracellular pH (Theparambil et al., 2020). Astrocytes can sense neuronal signals and, in response, regulate synaptic function and plasticity (for further review on this topic, see Araque et al., 2014; Bazargani and Attwell, 2016).

Processes of a single astrocyte can directly contact neuronal synapses, via peri-synaptic processes, as well as cerebral vessels, via endfeet processes; different subsets of proteins are enriched in each of these compartments (Stokum et al., 2021). The astrocyte-vessel interaction is extensive: an estimated 99.7% of brain vasculature is covered by astrocyte endfeet (Simard et al., 2003; Mathiisen et al., 2010). This polarity of astrocyte structure—their near-complete vascular ensheathment on the one hand and their close relationship with neurons on the other—allows astrocytes to relay signals between neurons and blood vessels in a bidirectional manner (Mishra, 2017; Presa et al., 2020). It is now well established that synaptic release of neurotransmitters (e.g., glutamate and ATP) can trigger increases in internal astrocyte Ca^2+^ (Porter and McCarthy, 1996; Winship et al., 2007; Mishra et al., 2016) and result in downstream release of vasoactive molecules onto the vasculature, thereby driving NVC (Zonta et al., 2003; Peng et al., 2004; Gordon et al., 2008; Mishra et al., 2016). A major class of astrocyte-derived vasoactive molecules include metabolites of arachidonic acid such as prostaglandin E2 (PGE2), epoxyeicosatrienoic acids (EETs), and 20-hydroxytetraenoic acid (20-HETE). PGE2 can have bidirectional effects on vascular tone depending on the receptor, however, in the cerebral circulation, it acts via the EP4 receptor on vascular mural cells to induce vasodilation (Davis et al., 2004; Hall et al., 2014; Mishra et al., 2016). EETs are known to be vasodilatory signals and act through the high-conductance Ca^2+^- and voltage-dependent K^+^ channel, B_K_, (Archer et al., 2003; Larsen et al., 2006), or by antagonizing the thromboxane receptor (Behm et al., 2009) on smooth muscle cells. 20-HETE is a strong vasoconstrictor that inhibits K^+^ currents, mainly through B_K_ channels, on smooth muscle cells, resulting in depolarization of VSMCs (Lange et al., 1997; Fan et al., 2013; Toth et al., 2013), but it may also act via the recently identified G-protein coupled receptor GPR75 (Garcia et al., 2017) or by increasing Ca^2+^ entry into vascular mural cells via L-type Ca^2+^ channels, TRPC6, and TRPV4 (Basora et al., 2003; Rousseau et al., 2005). Astrocytes can also release K^+^ ions via B_K_ channels on their endfeet, which can exert bidirectional effects on vessel tone by acting on various inwardly rectifying K^+^ channels depending on extracellular K^+^ concentration (Knot et al., 1996; Zaritsky et al., 2000; Filosa et al., 2006; Longden and Nelson, 2015). Astrocyte-derived ATP and its metabolite adenosine can also exert opposing effects on cerebral vessels, depending on the site of action (astrocytes, endothelial cells, or VSMCs) and the receptors involved. ATP generally constricts vessels (Kur and Newman, 2014; Kim et al., 2015) but, in some cases, it is also capable of dilating them (Horiuchi et al., 2001), potentially by enhancing the release of dilating agents from other nearby astrocytes (Wells et al., 2015) or endothelial cells (Toth et al., 2015). On the other hand, adenosine generally dilates vessels (Ngai and Winn, 1993; Gordon et al., 2008; Wang and Venton, 2017) but may also cause or modulate constrictions (Meno et al., 2001). In addition to NVC, astrocytes also play a role in maintaining the resting tone of the cerebral vasculature and set basal CBF levels (Kur and Newman, 2014; Rosenegger et al., 2015), which could affect the amplitude of activity-dependent CBF changes (Blanco et al., 2008).

Astrocytes can also signal in the opposite direction: they can receive signals regarding the overall physiological state of the body (e.g., blood pressure, CO_2_ levels, or osmolality) from blood vessels and, in turn, activate neurons to induce homeostatic neural control of peripheral organs (Gourine et al., 2010; Filosa et al., 2016; Wang and Parpura, 2018). Indeed, astrocytes seem capable of changing neuronal firing properties in response to changes in vascular flow and pressure (Kim et al., 2015, 2016). This novel concept, termed the vasculo-neuronal hypothesis (Moore and Cao, 2008; Filosa et al., 2016; Presa et al., 2020), may be a mechanism via which body state can dictate, or at least modulate, brain function.

Mature astrocytes vary in density by brain region (Emsley and Macklis, 2006) and display regional heterogeneity in morphology and gene expression (Farmer et al., 2016; Bayraktar et al., 2020). The intermediate filament protein GFAP is often used as an identifying marker for astrocytes but, as a structural protein, it is only useful in visualizing portions of the astrocyte cytoskeleton. For example, mature protoplasmic astrocytes within the gray matter have hundreds of fine spongiform processes that do not contain GFAP (Bushong et al., 2004). White matter astrocytes have thicker processes with typically denser GFAP and contact neurons most often at nodes of Ranvier (Bayraktar et al., 2014; Lundgaard et al., 2014). Hippocampal astrocytes also generally appear to have higher GFAP levels than cortical astrocytes. In addition to this difference in GFAP expression in different astrocyte populations, GFAP levels also change in a variety of contexts, including during development, aging, learning, and in response to injury (Arnold et al., 1996; Walz and Lang, 1998; Kamphuis et al., 2015; Huang et al., 2020). Multiple recent analyses show that astrocytes within the same general region can display sub-regional heterogeneity, for example in the cortex (Bayraktar et al., 2020) and cerebellum (Farmer et al., 2016), which likely reflects parcellation of function. Functional studies have also found that astrocytes in different sub-regions can have different mechanisms underlying similar intracellular signals; for example, Ca^2+^ transients in hippocampal stratum radiatum astrocytes depend on TRPA1 channels (Shigetomi et al., 2013), whereas in the stratum lucidum they do not (Haustein et al., 2014). More information on the regional variety of astrocyte properties and function, particularly in subcortical and brain stem regions, have yet to be discovered. Likewise, whether heterogeneity of astrocytes contributes to regional variations in neurovascular coupling (Ekstrom, 2021), such as that reported between the visual cortex and hippocampus (Shaw et al., 2021), is also unknown and a question that demands further attention.

### Microglia

Microglia originate very early in embryogenesis from yolk sac-derived myeloid precursor cells and infiltrate the brain by E8 (Alliot et al., 1999; Ginhoux et al., 2010). During development and continuing into the first week of life, microglia migrate along the cerebral vasculature to spread widely throughout the neuropil (Figure 2), where they proliferate and tile the tissue (Mondo et al., 2020).

Microglia are incredibly motile cells that extend and retract processes on the order of minutes to surveil their environment (Prinz et al., 2019). They frequently contact neurons, synapses, astrocytes, oligodendrocytes, and the microvasculature (Prinz et al., 2019), indicating an active role within the NVU. They play an important role in developmental refinement of neuronal circuits by engulfing dying neurons (Marin-Teva et al., 2004), pruning synapses (Stevens et al., 2007; Schafer et al., 2012; Stephan et al., 2012), and promoting mature, functional synapse formation (Ji et al., 2013; Pierre et al., 2017).

Within the NVU, microglia interdigitate with astrocytes to cover non-overlapping regions on CNS vessels (Mondo et al., 2020). This non-overlapping contact of the vasculature by astrocytes and microglia indicates that each cell type might have distinct roles within the NVU. Microglia play a crucial role in angiogenesis within the developing brain (Arnold and Betsholtz, 2013; Reemst et al., 2016). This vasculogenic role of microglia is underscored by the finding that microglia-deficient mice display impaired angiogenesis (Fantin et al., 2010). A recent study has also discovered an as-yet unappreciated role for microglia in regulating CBF; depletion of microglia or a microglia-specific knock-out of P2Y12R, a purinergic receptor characteristic of “resting” microglia in adult animals, resulted in a small but significant decrease in NVC and cerebrovascular reactivity (Császár et al., 2021, preprint). As monocyte-related cells, microglia also serve essential functions as the resident immune cells of the brain. Their processes dynamically surveil the brain microenvironment in healthy conditions (Madry et al., 2018). Following injury or in disease, microglia display a strong reactive response in which they retract their processes, reduce expression of resting phenotype proteins (such as P2Y12R, Cx3CR1, and TMEM119), and take on the amoeboid morphology and phagocytic characteristics of tissue macrophages (Lee et al., 2010; Salter and Stevens, 2017; van Olst et al., 2021). In this activated state, microglia can also release cytokines and other signaling molecules that induce reactive astrogliosis with a range of beneficial (Fukumoto et al., 2019) or detrimental (Liddelow et al., 2017) outcomes. Activated microglia also exert effects specific to the NVU, in particular by regulating the integrity of the BBB in a context-dependent manner (Halder and Milner, 2019; Haruwaka et al., 2019) and sometimes even engulfing segments of the vasculature (Jolivel et al., 2015). Thus, microglia play a multifaceted role in brain health. Although their association with blood vessels is established in both developing and mature brains, the exact role(s) of microglia within the NVU, in the context of the BBB as well as NVC, is underappreciated and has yet to be fully revealed.

### Vasculature

In humans, the large vessel architecture of the brain is established by birth (Streeter, 1921) though the intracerebral vascular network continues to grow during the initial part of postnatal development, with capillary angiogenesis (and new penetrating vessel formation, as necessary) matching cortical expansion to evenly supply the whole brain (Marin-Padilla, 2012). In rodents, the major pial arteries and penetrating arterioles are established during embryogenesis (Ruhrberg and Bautch, 2013; Shapiro and Raz, 2014; Coelho-Santos and Shih, 2020), but extensive growth and maturation of the microvasculature extend throughout the first weeks of life (Tata et al., 2015; Walchli et al., 2015a; Coelho-Santos and Shih, 2020). Highly branched capillary beds reach about 90% of total brain vessels, with proliferation sharply decreasing between P15-25, indicating maturation of the capillary structure around the third week of life (Harik et al., 1993). Astrocytes undergo massive proliferation and maturation within a similar timeframe, coinciding with postnatal angiogenesis and maturation of the capillary network. During vascular infiltration into the brain, the basal lamina of blood vessels and the astrocyte-secreted basal lamina of the brain merge to form an interconnected cerebrovascular basal lamina, to which both the vessels and the astrocyte endfeet are anchored (Figure 2; Marin-Padilla, 2012). This anchoring gives rise to the close physical relationship between these important NVU components and enables their concerted functions in BBB maintenance and NVC regulation.

All vasculature is composed of at least two main cell types—endothelial cells, which line the vessel wall and form the tube within which blood flows, and vascular mural cells, which are located abluminally on vessels, encircling the endothelial tube. During development, endothelial cells may also express GFAP, a protein associated primarily with astrocytes (Virgintino et al., 1993; Osman et al., 2020); thus, interpretation of endfeet coverage of the vasculature during development must be made using double labeling techniques to ensure that the endothelial tube is not being mistaken for astrocyte endfeet (Figure 1). Endothelial cells are crucial for maintaining the BBB, with their tight junctions forming the physical barrier and their expression of an array of transporters and trans-/para-cellular transport machinery tightly controlling the movement of substances across the BBB (Sweeney et al., 2019). Beyond the BBB, they are important for sensing mechanical signals in the blood, such as pressure/flow and shear stress, as well as endocrine signals, such as adrenaline and acetylcholine (Godo and Shimokawa, 2017). In response, endothelial cells can release substances to change the contractility of vascular mural cells, thus altering blood flow. For example, neuron-derived acetylcholine can increase intracellular Ca^2+^ in endothelial cells, triggering the production and release of NO onto VSMCs and driving vasodilation (Zuccolo et al., 2017). Shear stress can also induce Ca^2+^-dependent release of NO and prostacyclin from endothelial cells, both of which relax VSMCs, and dilate vessels (Hecker et al., 1993). The ability of endothelial cells to produce and release proteoglycans and glycoproteins to maintain the basement membrane and vascular integrity can also be altered by changes in intravascular pressure (for extensive review, see Tarbell et al., 2014). Upon sensing pathological signals, such as extreme shear stress, endothelial cells can secrete proteases to break up the extracellular matrix and induce vascular remodeling and neovascularization (Chatzizisis et al., 2007). Endothelial cells are interconnected via gap junctions, which allows them to propagate vasoactive signals from the nearby neuropil or vascular mural cells to adjacent endothelial cells, leading to conducted contractility changes. This allows local vascular networks to respond to mechanical or chemical stimuli as one unit (Hautefort et al., 2019). Endothelial cells may also play an active role in NVC (Hogan-Cann et al., 2019) by sensing signals from astrocytes and neurons and then releasing dilators like NO onto contractile vascular mural cells (Stobart et al., 2013; Zuccolo et al., 2017).

Vascular mural cells are divided into two main categories: VSMCs and pericytes. VSMCs are contractile mural cells that cover the endothelial cell layer on arterioles nearly completely and on venules to a lesser extent. In the brain, VSMCs on arterioles are important for the proper development of blood vessels (Hellstrom et al., 1999). Later, after the cerebrovascular network is established, their primary roles encompass maintaining resting vascular tone and enacting activity-dependent diameter changes during NVC. VSMCs can regulate resting tone of vessels throughout the body in response to autoregulation (flow/pressure changes) or when signaled by endothelial cells upon sensing shear stress. VSMCs in cerebral arterioles can also regulate tone in response to signals from astrocytes via at least two mechanisms: purinergic signals from astrocytes constrict arterioles (Kur and Newman, 2014; Kim et al., 2015) while prostaglandins from astrocytes dilate arterioles (Rosenegger et al., 2015). Together, these push-and-pull signals establish a healthy vascular tone. On top of this basal regulation, VSMCs also participate actively in NVC by relaxing or contracting swiftly in response to neuronal activity to change arteriole diameter and produce changes in CBF (Attwell et al., 2010; Iadecola, 2017; Mishra, 2017). This VSMC response can be induced by direct signals from neurons, such as prostaglandins (Lacroix et al., 2015) or NO (Lourenço et al., 2014; Mishra et al., 2016; Echagarruga et al., 2020) or in response to astrocyte-derived signals, such as PGE2, EETs, 20-HETE, and K^+^ (Peng et al., 2002; Mulligan and MacVicar, 2004; Filosa et al., 2006; summarized in Iadecola, 2017).

Pericytes are vascular mural cells that ensheath the capillary endothelial tube. They are easily identified by the morphology of their cell bodies, which are situated on the vessel with a “bump-on-a-log” morphology, with processes extending along and around capillaries (Attwell et al., 2016). Pericyte morphology is dependent on their location on the vascular tree: pericytes on the first capillaries branching from arterioles have a more circumferential nature with a meshwork of processes around the vessel, while those in the mid-capillary network have more elongated longitudinal processes and those closer to the venules take on almost an amoeboid mesh morphology (Hartmann et al., 2015). At least at the level of the first capillary branches coming off the penetrating arterioles, pericytes mediate precise and local changes in vessel diameter and therefore are prime candidates for controlling localized, microvascular blood flow changes (Hall et al., 2014; Cai et al., 2018; Rungta et al., 2018). These pericytes can control blood flow by changing capillary diameter in response to neurotransmitters such as noradrenaline and glutamate (Hall et al., 2014) or in response to local neuronal activity (Mishra et al., 2016; Cai et al., 2018; Rungta et al., 2018). In mid-capillary regions, contraction of the longitudinal processes of pericytes is likely to increase rigidity, thus decreasing pliability, of the endothelial tube. As capillaries are generally narrower than red blood cells, such a change in pliability could potentially have large effects on net red blood cell flux. Thus, given that capillaries have the greatest resistance in the cerebrovascular system, changes in capillary diameter or pliability are likely to be a major source of blood flow changes in the brain (Gould et al., 2017).

Pericytes are functionally coupled to endothelial cells via gap junctions in both rodents and humans (Cuevas et al., 1984; Kovacs-Oller et al., 2020). Endothelial cells are tightly coupled together by gap junctions (Hautefort et al., 2019) to allow electrotonic conduction along the vasculature (Segal and Duling, 1986; Welsh and Segal, 1998; Emerson and Segal, 2000). This relationship permits locally sensed contractile signals in one pericyte to be conducted along the endothelium and conveyed to distant pericytes (Wu et al., 2006) or upstream arterioles (Longden et al., 2017). This mechanism allows propagation of vascular responses up- and down-stream of the vasculature to mount a coordinated response to increase local CBF within a capillary network (Segal and Duling, 1986; Rungta et al., 2018; Kovacs-Oller et al., 2020).

## Neurovascular Coupling Changes in Development

The NVC relationship changes dramatically during development. In humans, BOLD-fMRI studies show that infants often display sensory stimulation-coupled negative BOLD signals at higher rates and positive BOLD response less often than adults (Martin et al., 1999). Interestingly, newborns have a positive BOLD response but the negative BOLD response develops after birth, around 8 weeks of age (Altman and Bernal, 2001), peaks sometime during the second to third year of life, and decreases thereafter until a stable mature positive BOLD relationship is established (Martin et al., 1999).

The BOLD signal arises from a balance of oxygenated and deoxygenated blood and is influenced by changes in both CBF and metabolism (Howarth et al., 2021). Deoxygenated hemoglobin has paramagnetic properties; therefore, if metabolism alone were to increase (oxygen consumption), the increase in deoxyhemoglobin would actually cause the BOLD signal to decrease. However, NVC results in an increase in CBF in active brain regions, thus washing out deoxyhemoglobin and perfusing the tissue with an oversupply of oxyhemoglobin. This results in the positive BOLD signals (Ogawa et al., 1998; Howarth et al., 2021). Thus, negative BOLD responses could result from either an overburden of metabolism despite a comparable increase in CBF or an absence of activity-coupled CBF increase. The negative BOLD signals in human infants coincide with a developmental period of rapid synapse formation (Huttenlocher et al., 1982); thus, one possible explanation is that the increase in metabolic demand of the neurons is not matched by CBF (Meek et al., 1998). As synapse pruning and elimination proceed during the first several years of human life (Huttenlocher et al., 1982), the metabolic demand is reduced and NVC can match, and indeed out-supply, oxygen use. However, there is also evidence to suggest that the negative BOLD in neonates might be due to a smaller increase or even decrease in CBF in response to neuronal activity. Resting CBF values are lower in neonates and reach adult levels during adolescence (Takahashi et al., 1999), reflecting the time course of the still-growing cerebrovasculature (Marin-Padilla, 2012). Furthermore, a side-by-side comparison of ages showed a visual stimulation-evoked *decrease* in both CBF and BOLD signals in children, while an increase in both parameters was detected in adults (Born et al., 2002). Thus, both an increased metabolic demand and decreased NVC may together contribute to the negative BOLD signal in infant humans.

A similar switch in NVC is also observed in rodents. A systematic study in rats showed that hind paw stimulation evokes a decrease in CBF in the somatosensory cortex of young rats up to P18 but switches between P21–23 to become an increase in CBF and is strengthened even further in adulthood (Kozberg et al., 2013). In this study, total hemoglobin levels were used as a proxy measure of CBF, which should reflect the combination of both deoxy- and oxyhemoglobin and thus reflect overall changes in CBF. The observed pattern matches the findings of BOLD studies in human infants (interspecies age-corrected) and further supports the idea that a decrease in CBF, rather than an increase in metabolic oxygen use alone, contributes to negative BOLD. Thus, the first three weeks of rodent life are characterized by a correlated growth of the vascular network, astrocyte proliferation and maturation, and the establishment of a healthy mature NVC response. Although incomplete, a comparable picture exists in humans.

Astrocyte-mediated changes in vascular diameter are widely recognized as a major mechanism regulating vascular tone and NVC and are expected to contribute significantly to changes in CBF (Mishra, 2017). In response to neural activity, astrocytes can regulate the diameter of cerebral vessels in a Ca^2+^-dependent manner via several mechanisms. They can synthesize and release arachidonic acid metabolites such as EETs and PGE2 to dilate vessels, or 20-HETE to constrict vessels. They also take up excess extracellular K^+^ during neuronal activity, which depolarizes them and leads to Ca^2+^ increases via an as-yet unknown mechanism. This ultimately activates B_K_ channels and releases K^+^ onto vascular mural cells (Filosa et al., 2006). Depending on the amount of K^+^ released, vascular cells can hyperpolarize (up to ∼15 mM) or depolarize (>20 mM), thus producing vascular dilation or constriction, respectively (Girouard et al., 2010). Astrocytes can also release ATP directly onto vascular cells in a tonic manner (Kur and Newman, 2014) or in response to increases in intravascular pressure (Kim et al., 2015). ATP can act on P2X receptors on the vascular cells to constrict them or it can be converted by extracellular ectonucleotidases to adenosine, which dilates vessels (Gordon et al., 2008). ATP from astrocytes can also drive vasodilation through ATP-mediated activation of endothelial NO synthase (eNOS) via P2Y_1_ receptors (Toth et al., 2015). Astrocytes and glial cells with similar properties (such as retinal Müller glia and cerebellar Bergmann glia) have been shown to release ATP in a Ca^2+^-dependent manner (Xiong et al., 2018), however, this has yet to be established in the context of NVC.

Most data support the idea that many astrocyte-dependent NVC mechanisms are, or theoretically could be, regulated by intracellular Ca^2+^ level within astrocytes. Two concepts have remained controversial: firstly, the source of the Ca^2+^ signals within astrocytes, and secondly, whether astrocyte engagement results in dilation or constriction. We suggest that the seemingly conflicting observations reported by different groups reflect developmental changes in NVC and therefore might offer a more nuanced insight into NVC maturation mechanisms than is appreciated. The first study to suggest a role for astrocytes in NVC was performed in adult rats and showed that a glutamate-evoked increase in CBF measured by laser Doppler flowmetry relied on the release of EETs from astrocytes (Alkayed et al., 1997; Peng et al., 2004). Later, metabotropic glutamate receptor 5 (mGluR5) dependent increases in astrocyte Ca^2+^ signals were found to underlie neural activity-evoked arteriole dilation in the cortex of P9–15 rats (Zonta et al., 2003). Although only the mechanism of dilations was probed in this study, only half of the vessels studied dilated, suggesting that the other half either did not respond or constricted. Another study performed in the hippocampus of P13–18 rats and mice showed that exogenously increasing astrocyte Ca^2+^ by activating mGluR5 or uncaging Ca^2+^ evoked arteriole constrictions (Mulligan and MacVicar, 2004). Thereafter, an *in vivo* study found that activity-evoked increases in CBF depended, at least partly, on mGluR5 in two-month-old mice (Takano et al., 2006). Thus, although it seemed that mGluR5-driven Ca^2+^-dependent mechanisms in astrocytes played a prominent role in cerebrovascular regulation, the response evoked was inconsistent, sometimes resulting in dilations (or an increase in CBF) and sometimes constrictions.

It soon emerged that mGluR5 expression in astrocytes decreases as early as the second postnatal week and is essentially absent in adults (Rusnakova et al., 2013; Sun et al., 2013; Clarke et al., 2018). Indeed, mGluR5 had previously been shown to be functionally absent in astrocytes in P21 rat hippocampal slices as early as 1995 (Duffy and MacVicar, 1995), and it was shown to decrease at the protein level with age and disappear between P20–25 in 2000 (Cai et al., 2000). Thus, much of the work showing a role for astrocytic mGluR5 in NVC may entirely be a factor of developmental age. In line with this, at least two studies performed in rats older than P21 showed no effect of mGluR5 inhibition on NVC, either *in vivo* (Calcinaghi et al., 2011) or *in vitro* (Mishra et al., 2016). Furthermore, mGluR5 is expected to increase Ca^2+^ in astrocytes via release from internal stores in an inositol trisphosphate (IP3) receptor (IP3R)-dependent manner. However, mice older than P45 lacking IP3R2, which is the primary IP3R type in astrocytes, failed to block both neurally evoked arteriole dilation (Bonder and McCarthy, 2014) and BOLD signals (Jego et al., 2014), further supporting the idea that NVC in adults does not rely on astrocytic mGluR5 or indeed any Gq-coupled internal store dependent Ca^2+^ release mechanism. New evidence suggests that astrocyte Ca^2+^ can be raised by several channels and receptors in an IP3-independent manner (Bazargani and Attwell, 2016). One such mechanism appears to involve neuron-to-astrocyte ATP-dependent signaling, which activates P2X1/5 in astrocytes and causes Ca^2+^ influx across the plasma membrane from the extracellular space (Lalo et al., 2008). This purinergic signaling pathway via P2X1 was shown to be a key pathway in astrocyte-mediated NVC at the capillary level (Mishra et al., 2016). It is likely these are P2X1/5 receptors, as the antagonists used in this study inhibits both the homotrimeric P2X1 and heterotrimeric P2X1/5 receptors, and the kinetics of the evoked Ca^2+^ signals were reflective of the heterotrimer.

Thus, during development, astrocytes are associated with both activity-dependent vasodilation and, more often, vasoconstriction. This variability in response may underlie the decrease in CBF and BOLD signals during early development. By adulthood, astrocyte-mediated NVC rarely results in vasoconstriction. Therefore, the consistent and reliable functional hyperemia observed in adults appears to be driven by maturation of the NVU system and NVC mechanisms. The specific mechanisms underlying these differences are not known, although a clue might be in the amount of astrocyte Ca^2+^ produced in response to neural activity at different ages. Large increases in astrocyte Ca^2+^ can drive constrictions, while more moderate amounts tend to drive dilations (Girouard et al., 2010). The type and amplitude of astrocyte Ca^2+^ signal evoked by neural activity are determined by the type of astrocyte receptor activated. Being a cation channel, P2X1 (or P2X1/5) receptor activation may produce a relatively small increase in astrocyte Ca^2+^, whereas the developmental pathway engaging mGluR5 would, due to the nature of Gq-coupled Ca^2+^ signals, induce a large increase in astrocyte Ca^2+^. Could such differences in the mechanism and size of astrocyte Ca^2+^ signal generation be responsible for developmental differences in NVC? This is a question that has yet to be conclusively answered. However, some evidence in support of the idea exists: rapid and short-latency Ca^2+^ responses *in vivo* are correlated with functional hyperemia (Winship et al., 2007) and appear to enhance the early hemodynamic response (Gu et al., 2018; Tran et al., 2018). On the other hand, slower Ca^2+^ responses may be more reliable for modulating resting tone (Kur and Newman, 2014; Kim et al., 2015; Rosenegger et al., 2015) or inducing vessel constriction following hyperemia to restore basal CBF (Gu et al., 2018; Tran et al., 2018). Furthermore, resting vessel tone itself can also influence the polarity of the response evoked by vasoactive stimuli, whereby more constricted vessels exhibit a larger dilatory response (Blanco et al., 2008). How strong of a role such an indirect modulatory function of astrocyte-dependent vascular tone could play in the developmental maturation of NVC is unknown.

## Neurovascular Coupling in Disease

The evolutionary conservation of NVC in vertebrates and the robustness of the response indicate that it is required to support healthy brain metabolism. The increase in CBF in response to neural activity is also suggested to play a role in clearing the brain of waste by-products of normal function and metabolism, either via the blood itself or via perivascular mechanisms (Iliff et al., 2012; Ramanathan et al., 2015; Bacyinski et al., 2017). Such by-products include, among others, CO_2_, amyloid-β (Aβ), and tau (Sagare et al., 2007; Iliff et al., 2012; Dadas et al., 2016; Kisler et al., 2017a). A decrease in CBF or a loss of normal, robust NVC could theoretically starve neurons of necessary energy requirements while also creating a toxic environment due to lack of clearance. Reduction in CBF or diminished NVC are common clinical features of various neurological disorders such as multiple sclerosis (MS), spinal cord injury, traumatic brain injury, stroke, and several types of dementias including AD and AD-related mixed dementias (Joo Turoni et al., 2011; Lin et al., 2011; Tarantini et al., 2017; Stickland et al., 2019; Sachdeva et al., 2020; Sharma et al., 2020). It is clear that NVC is a component of healthy brain function, but whether NVC deficits drive pathology or if pathology drives NVC deficits is largely unknown and needs more focused study.

In this section, we will cover NVC dysregulation in two specific contexts: stroke and AD-related dementias. Of importance, patients with AD are more likely to have a history of silent brain infarcts and microinfarcts, while patients with strokes or even transient ischemic attacks have increased incidence and progression of dementia (Snowdon et al., 1997; Kalaria, 2000; Miklossy, 2003; Vermeer et al., 2003, 2007; Yang et al., 2015; Korte et al., 2020). Although the link between stroke and dementia is not completely understood, diminished NVC in both instances could be a clue. In fact, most cases of dementia are classified as mixed dementias that present most commonly with a pure AD component compounded by vascular impairments, often due to ischemic injuries (Kapasi et al., 2017; Gladman et al., 2019). Another common factor in neurological disease including AD and stroke is reactive astrogliosis —a response of astrocytes to non-physiological changes in their microenvironment (Sofroniew, 2020). This response produces myriad morphological, transcriptional, and functional changes within astrocytes over days and weeks in a context dependent manner (Sofroniew, 2009; Zamanian et al., 2012). In a study comparing stroke (middle cerebral artery occlusion, MCAO) and infection-related inflammation (intraperitoneal lipopolysaccharide, LPS) models, transcriptional changes in astrocytes differed starkly by paradigm (Zamanian et al., 2012). For example, increases in intermediate filament proteins like GFAP and vimentin were increased in both conditions, but nestin, another intermediate filament protein, was only increased in the MCAO model. Overall, this study identified 263 genes whose expression was significantly induced by at least 4-fold 1 day after injury with 206 after MCAO, 113 after LPS, and 56 in both conditions. These widespread transcriptional changes could alter astrocyte function; however, little is known about such functional consequences, especially in the context of vascular regulation (McConnell et al., 2019).

### Stroke

Nearly 800,000 patients suffer a stroke in the United States every year. Approximately 87% of these are ischemic strokes, where blood flow to neural tissue is blocked or reduced, with the remaining comprised of hemorrhages (intracerebral, intraventricular, and subarachnoid) (Virani et al., 2020). In ischemic strokes where a blood vessel is blocked by a clot or emboli, recanalization of the vessel alone is insufficient for positive prognosis (Levine et al., 2015) and a decrease in CBF in the stroke adjacent tissue, determined as a decrease in capillary flow, predicts worse outcomes (Jagani et al., 2017). Ischemic strokes may also occur following stenosis of a large artery in the absence of a clot. If the stenosis occurs in a vessel supplying a territory with poor collateral flow, typically called “watershed” territories, the drop in blood pressure distal to the stenosis may reduce blood flow to such an extent that ischemic injury ensues (Naritomi et al., 1985). Following hemorrhagic stroke also, the disruption in blood flow by the bleed can give rise to ischemic conditions in the downstream territory of the vessel. In both ischemic and hemorrhagic strokes, vascular dysfunction is clinically well established. Diminished NVC is observed in ischemic stroke patients for at least up to a decade following initial infarct (Krainik et al., 2005; Lin et al., 2011). This long-term impairment was evidenced by a variety of techniques (laser Doppler flowmetry, cerebral angiography, and BOLD), underscoring the robustness of the finding. It is also widespread, such that multiple regions distant from the infarct exhibit diminished NVC (Krainik et al., 2005). Following subarachnoid hemorrhage, an adverse event termed delayed cerebral ischemia can occur in a subset of patients. Although this later ischemic event was traditionally believed to be caused by delayed vasospasm of cerebral arterioles, recent evidence has failed to support this idea (Koide et al., 2012). Instead, other factors such as neuroinflammation, cortical spreading depolarization, and NVC defects have been proposed to play a more prominent role (Dreier, 2011). Cortical spreading depolarizations are common after all types of stroke and evidence suggests that under non-physiological conditions, this massive depolarization wave is concurrent with a negative NVC, such that neuronal depolarization is accompanied by a decrease in blood flow (Sukhotinsky et al., 2008) and an increase in vasoconstriction (Koide et al., 2012; Pappas et al., 2015). It should be noted that an absence of NVC may also precipitate if the vasculature is fully dilated as an adaptive mechanism to cope with lack of blood flow, particularly in regions with few collateral vessels. If this is the case, the lack of NVC would not really be a loss of function, rather it may be interpreted as having reached its maximum capacity. In such cases, the only way to restore NVC may be reestablishment of normal vascular tone following treatment of the underlying cause (for example, stenosis). This important distinction between lack of dilation and presence of maximum dilation at baseline must be resolved in the various stroke conditions despite their surface similarities.

Following stroke, both glial cells (microglia and astrocytes) and the vasculature change drastically; these changes may contribute to long term stroke-induced neurodegeneration and dementia (Lin et al., 2011). Brain injuries such as ischemic stroke contributes to microglia activation, and activated microglia can promote injury or promote repair, depending on the activation signals (Ma et al., 2017). Activated microglia can produce pro-inflammatory mediators such as tumor necrosis factor α, interleukin-1β, NO (via inducible NO synthase), and multiple proteolytic enzymes (Yenari et al., 2010), as well as tissue-restoring signals such as interleukin-10, transforming growth factor β, insulin-like growth factor, and vascular endothelial growth factor (Ponomarev et al., 2013). Microglia quickly respond to ischemic stroke by accumulation within the lesion and persist in the region for at least 7 days (Perego et al., 2011). The dual role of microglia is evident at the post-ischemic NVU; microglia may enhance BBB integrity after stroke by clustering around blood vessels and secreting pro-angiogenic molecules such as vascular endothelial growth factor (Welser et al., 2010; Zhao et al., 2018), but they may also enhance degeneration of the NVU by engulfing the vasculature, thus allowing blood proteins to leak into the brain (Jolivel et al., 2015).

Among vascular cells, capillary pericytes display a strong response to ischemic insults. Even after the clot is removed from the blocked artery and the vasculature is recanalized, perfusion through the capillary network remains perturbed (Jagani et al., 2017). This reduced capillary perfusion is attributed to narrowing of the capillary lumen, potentially due to “extrinsic compression” or obstruction of flow by platelet aggregation (micro-clots) or leukocyte adhesion (del Zoppo and Mabuchi, 2003). More recently, pericytes were observed to contract in response to ischemia and therefore constrict capillaries (del Zoppo and Mabuchi, 2003; Yemisci et al., 2009; Hall et al., 2014). This results in inadequate tissue reperfusion that can exacerbate stroke response even when reperfusion is established (Dalkara et al., 2011).

Following ischemic injury, glial scar formation by astrocytes is crucial for repairing the BBB, minimizing neuron cell death and limiting invading immune cells into the brain (Sofroniew, 2009). However, the role of reactive astrocytes in the long-term health of the brain is still in debate. Transcriptional changes in reactive astrocytes following experimental models of stroke are detectable 24 h after ischemia and persist for at least 14 days (Kindy et al., 1992; Zamanian et al., 2012; Rusnakova et al., 2013). Interestingly, transcriptional changes in these astrocytes include the typical markers of reactive astrogliosis such as increased GFAP, but also recapitulate many characteristics associated with immature astrocytes, such as vimentin, nestin, Cspg4, and Pdgfra (Zamanian et al., 2012; Rusnakova et al., 2013). One analysis of the reactive astrocyte transcriptome following an MCAO model of stroke identified a total of 263 individual genes that increased at least fourfold after injury (Zamanian et al., 2012). Another study examined a targeted subset of transcripts within reactive astrocytes following the same model of stroke and identified expression changes showing a reasonable overlap with the former study (Rusnakova et al., 2013). The differentially upregulated genes in stroke-induced reactive astrocytes included calcium binding proteins (e.g., S100b), glutamate transporters (e.g., Eaat1) and receptors (e.g., Gria2, Grin2a, Grin2b, and Grm5), potassium channels (e.g., Kcnj10 and Hcn2) and water homeostasis channels (e.g., aquaporin 4) (Rusnakova et al., 2013). These transcriptional changes could greatly alter the function of astrocytes over time. Importantly for NVC signals, upregulation of various pathways that give rise to astrocyte Ca^2+^, particularly metabotropic neurotransmitter receptors, are evident and persistent. Indeed, following subarachnoid hemorrhage, the inversion of NVC (meaning that neuronal activity produces vasoconstriction rather than dilation) depends on enhanced Ca^2+^ signaling in astrocytes leading to increased K^+^ release at the vasculature (Pappas et al., 2015). A potent vasoconstrictor, 20-HETE, was detected at significantly higher levels in the plasma of ischemic stroke patients and CSF of subarachnoid hemorrhage patients (Crago et al., 2011; Yi et al., 2017), which may also partially be responsible for diminished NVC. 20-HETE is a metabolite of arachidonic acid, which is largely synthesized by astrocytes in the brain, leading us to speculate that an astrocyte-dependent pathway may play a role. Further, the upregulation of various ionotropic and metabotropic glutamate receptors in astrocytes after stroke also recapitulates some of their developmental characteristics. In particular, the metabotropic receptor mGluR5 is also upregulated in reactive astrocytes in animals models of demyelination (Jean et al., 2008; Planas-Fontanez et al., 2020) and epilepsy (Aronica et al., 2000; Ulas et al., 2000; Ferraguti et al., 2001). Could these expression changes contribute to increased depolarization of the astrocyte membrane and larger Ca^2+^ signals from internal stores, altering the NVC response? We hope this tantalizing question attracts much future scientific investigation.

### Alzheimer’s Disease and Related Dementias

Diminished NVC is seen with age, however, it is accelerated in the context of dementia and AD (Park et al., 2007; Toth et al., 2014; Tarantini et al., 2017). AD is the most common form of dementia, first described by Alois Alzheimer in 1907. It is characterized by extracellular Aβ aggregations in the neuropil and around blood vessels, and deposition of fibrillary tau protein aggregates in neurons (Alzheimer, 1907; Kosik et al., 1986; Goedert et al., 1988; Selkoe and Schenk, 2003). In AD, the hallmark loss of neurons is accompanied by a loss of microvessels, endothelial cells, and smooth muscle cells, indicating a close link between neuronal loss and cerebrovascular changes (Farkas and Luiten, 2001). Vascular disorders such as hypertension and arteriosclerosis have also been identified as risk factors for cognitive impairment and decline (Shapiro et al., 1982; Scherr et al., 1991). Indeed, AD most commonly manifests alongside other pathologies, which has given rise to the concept of mixed dementias. The most common mixed dementia type possesses the symptoms of AD combined with vascular dysfunction (Kapasi et al., 2017; Gladman et al., 2019). Loss of neurovascular coupling is prevalent in AD and recapitulated in animal models of the disease (Iadecola, 2013; Tarantini et al., 2017). Although the relationship between AD and vascular dysfunction is widely recognized, whether this is a causative or a correlative relationship is not fully understood.

Cerebrovascular dysfunction is an early event in individuals who develop cognitive impairment and progress to dementia (Iturria-Medina et al., 2016). In mouse models of AD, overexpression of amyloid precursor protein (APP) can drive Aβ deposits, and these deposits have been associated with cerebrovascular dysregulation (Zhang et al., 1997; Koistinaho et al., 2002), decreased NVC (Niwa et al., 2000b) and increased microvessel response to vasoconstrictor molecules, potentially biasing the vasculature toward constriction over dilation (Niwa et al., 2001). Aβ can directly constrict capillaries by engaging endothelin, a strong vasoconstrictor, and downstream of oxidative stress (Nortley et al., 2019). Aβ deposition around parenchymal arterioles, a common feature of AD, may also stiffen the blood vessels and reduce their ability to respond to endogenous vasoactive substances (Kimbrough et al., 2015). Although tau mutations have not thus far been associated with AD itself, animal models with mutated tau forms that give rise to neurofibrillary tangle pathology similar to AD show a reduction in NVC (Park et al., 2020). Furthermore, these vascular dysfunctions can occur prior to changes in energy utilization or neuronal function (Park et al., 2020). This mismatch in energy utilization and supply occurs early and is likely to be causative in disease progression; however, empirical data to support this hypothesis are still weak.

Neurovascular coupling changes after ischemia or in AD could be the result of impaired activity of neurons, astrocytes, or vasculature; likely, it is a combination of all three. Although neuronal dysfunction in AD has been extensively investigated over the last century, studies exploring changes in astrocyte and vascular function have only begun in the past two decades and we have yet a lot to learn about how these components of the NVU contribute to pathology. Indeed, pericytes have recently emerged as a key contributor to AD-related vascular damage. Pericyte degeneration is evident in AD patients, contributing to a decrease in vessel motility and likely influencing neurovascular coupling (Halliday et al., 2016). Transgenic mice in which pericyte degeneration is induced have a leaky BBB, reduced CBF, and diminished NVC, all of which precede neurodegenerative changes (Kisler et al., 2017b, 2020; Montagne et al., 2018). Prominent changes are also observed in glial cells – both microglia (Venneti et al., 2008; Hansen et al., 2018) and astrocytes (Phatnani and Maniatis, 2015) become reactive in AD with several significant transcriptional changes. As a key vascular regulator in the brain, astrocytes that are reactive are likely to have an effect on both basal vascular tone and NVC in AD. Functionally, increased astrocyte Ca^2+^ signaling has been observed in AD (Kuchibhotla et al., 2009). As the amplitude of Ca^2+^ signals in astrocytes can tune the polarity of the vascular response (Girouard et al., 2010), such observations entice one to conjecture that their role in NVC must be altered. Vascular intrinsic events such as neutrophil adhesion to the capillary endothelial tube may also obstruct the flow of blood and contribute to reduced CBF (Cruz Hernandez et al., 2019). These findings indicate that in addition to neuronal loss, a vast number of changes in the individual components of the NVU could drastically alter NVC and CBF in the context of AD. This is a field ripe for future research ventures.

The recently described glymphatic pathway for perivascular removal of extracellular substances may also be compromised in AD and AD-related dementias (Iliff et al., 2012). This mechanism is important in clearing Aβ (Iliff et al., 2012) and potentially also tau protein (Iliff et al., 2014; Harrison et al., 2020). Moreover, the glymphatic waste clearance pathway is more active during sleep (Xie et al., 2013) and occurs in an astrocyte AQP4-dependent manner (Iliff et al., 2012; Mestre et al., 2018). A decrease in sleep quality (Ju et al., 2013) and changes in astrocytic AQP4 expression and polarization (Boespflug et al., 2018) are observed in AD. Changes in vascular tone can also actively change the perivascular space volume and alter glymphatic flow, as shown following stroke (Mestre et al., 2020). Changes in astrocyte functions after stroke or in AD-related dementias could drive impaired perivascular clearance, either directly due to altered expression of AQP4 or other genes involved in this process or indirectly by altering vascular tone, and therefore contribute to AD. This hypothesis has been proposed (Braun and Iliff, 2020) but remains to be rigorously tested.

## Conclusion

The close coordination of neurons, glia, and vasculature is integral to CBF regulation and therefore healthy brain function. The differences between NVC in early versus late developmental stages are striking and require extensive future research to understand the underlying molecular differences at the level of neurons, glia, and vasculature. Similarly, while dysregulation of NVC is observed in disease and injury contexts such as stroke and AD, the precise molecular mechanisms underlying these changes remain unknown. With the noted similarities in NVC and astrocytes between development and disease, a deeper understanding of NVC in the context of the former may inform the latter.

## Author Contributions

All authors listed have made a substantial, direct and intellectual contribution to the work, and approved it for publication.

## Conflict of Interest

The authors declare that the research was conducted in the absence of any commercial or financial relationships that could be construed as a potential conflict of interest.
